# Herpesvirus reactivation is associated with mortality in critically ill ICU patients with COVID-19: Insights from a retrospective single-center analysis of 455 cases

**DOI:** 10.1371/journal.pone.0354153

**Published:** 2026-07-17

**Authors:** Stefanie Michel, Cilian Kempf, Nina Pirschtat, Frank Herbstreit, Bettina Budeus, Sebastian Voigt, Jutta Dedy, Thorsten Brenner, Simon Dubler

**Affiliations:** 1 Department of Anesthesiology and Intensive Care Medicine, University Hospital Essen, University of Duisburg-Essen, Hufelandstraße, Essen, Germany; 2 Institute of Cell Biology (Cancer Research), University of Duisburg-Essen, Hufelandstraße, Essen, Germany; 3 Institute for Virology, University Hospital Essen, University of Duisburg-Essen, Hufelandstraße, Essen, Germany; 4 Pharmacy, University Hospital Essen, University of Duisburg-Essen, Hufelandstraße, Essen, Germany; University of St Andrews, UNITED KINGDOM OF GREAT BRITAIN AND NORTHERN IRELAND

## Abstract

**Background:**

Critically ill patients with COVID-19 frequently develop viral reactivation, yet the clinical significance of herpes simplex virus type 1 (HSV-1) and cytomegalovirus (CMV) remains a matter of debate.

**Methods:**

We conducted a retrospective single-center study of 455 consecutive intensive care unit (ICU) patients with confirmed SARS-CoV-2 infection between March 2020 and December 2021. Patients underwent screening for HSV-1 and CMV in whole blood and respiratory samples. A multivariable model was used to independently investigate risk factors associated with mortality.

**Results:**

The mean age was 57 years (range: 13–94 years), and 316 (69%) were male. During a median ICU stay of 14 (0–90) days, viral reactivation (either HSV-1 or CMV or both) occurred in 164 (36%) patients. HSV-1 and CMV co-reactivation was detected in 57 (13%) patients, isolated HSV-1 reactivation was observed in 54 (12%) patients and isolated CMV reactivation in 53 (12%) patients. Median time from ICU admission to viral detection was 4 days, with a stepwise increase with longer ICU stay. Detection of HSV-1 was associated with decreased ICU survival (32% versus 67%; p < 0.001) which remained significant after multivariable adjustment. Detection of HSV-1 but not CMV was associated with an increased need for invasive mechanical ventilation, veno-venous extracorporeal membrane oxygenation therapy, and the need for renal replacement therapy. Higher neutrophil-lymphocyte ratios were independently associated with an increased risk of ICU mortality and correlated with detection of HSV-1 and CMV.

**Conclusion:**

Herpesvirus reactivation was common in critically ill patients with COVID-19. HSV-1 reactivation was associated with a more severe course of disease and increased ICU mortality.

## Background

The COVID-19 pandemic was a leading cause of intensive care unit (ICU)-related mortality, exerting unprecedented pressure on healthcare systems worldwide [1]. Although the development and widespread implementation of vaccination programs led to a significant reduction in morbidity and mortality, SARS-CoV-2 infections remain clinically relevant. Vulnerable patient groups include older adults, immunocompromised individuals, and those with comorbidities. ICU mortality in these subgroups remains considerable [2]. Various host-related risk factors with poor outcomes in COVID-19 have been identified; however, predicting the individual disease trajectory of critically ill patients remains challenging [3]. Given the dynamic interplay of viral replication, immune dysregulation, and secondary infections, timely identification of high-risk patients is crucial for therapeutic decision-making and resource allocation in the intensive care setting.

Detection of viruses other than SARS-CoV-2 is common in critically ill patients with COVID-19 and is further promoted by corticosteroid therapy, broad-spectrum antibiotics, and invasive mechanical ventilation [4,5]. In particular, herpesvirus reactivations, including herpes simplex virus type 1 (HSV-1) and cytomegalovirus (CMV), are frequently detected in ICU patients, with conflicting results regarding their impact on outcomes in critically ill individuals with COVID-19 [6–8]. Accordingly, evidence-based guidance on when to initiate antiviral therapy in these vulnerable patients is limited.

The aim of this study was to assess the impact of HSV-1 and CMV reactivation on disease characteristics, progression, treatment, and prognosis in critically ill patients with COVID-19 requiring intensive care treatment. In a large single-center dataset including 455 critically ill COVID-19 patients, we demonstrate high rates of HSV-1 and CMV reactivation with an adjusted association of HSV-1 with adverse outcome and increased ICU mortality (**Supplementary** S2 Fig).

## Methods

We conducted a single-center retrospective study on herpesvirus reactivation as a risk factor for mortality in critically ill patients with COVID-19. Data were accessed and collected for research purposes between 01/07/2025 and 28/02/2026, followed by subsequent data analysis. All consecutive patients with a confirmed positive test for SARS-CoV-2 between March 2020 and December 2021 who were treated at the anesthesiology ICU of University Hospital Essen, Germany, were screened for inclusion. SARS-CoV-2 was detected by polymerase chain reaction (PCR) using nasopharyngeal swabs in spontaneously breathing patients and bronchoalveolar lavage fluid (BALF) in invasively mechanically ventilated or tracheotomized patients. In all patients with acute respiratory distress syndrome (ARDS), CMV serostatus (IgG/IgM) and a CMV PCR assay were routinely obtained at hospital admission according to local protocol [9]. Thereafter, CMV PCR testing in whole blood was performed once weekly. Testing for HSV-1 in BALF was indicated in invasively mechanically ventilated or tracheotomized patients, as well as in patients receiving continuous positive airway pressure/non-invasive ventilation in cases of extubation failure, weaning failure, or clinical suspicion of ventilator-associated pneumonia. Pneumonia was defined in accordance with the guidelines of the German Respiratory Society [10] as the presence of new or progressive pulmonary infiltrates on chest radiography or computed tomography, combined with at least one clinical criterion such as fever or hypothermia, cough with purulent sputum, dyspnea or tachypnoea, focal auscultatory findings, or systemic inflammatory markers, including alterations in C-reactive protein (CRP), leucocyte count, or procalcitonin. The cohort included not only patients with COVID-19 pneumonia, but also patients in whom SARS-CoV-2 infection was incidental. Sepsis was defined as life-threatening organ dysfunction caused by a dysregulated host response to infection with sepsis-associated organ dysfunction being diagnosed by an increase in the Sequential Organ Failure Assessment (SOFA) score of ≥2 points [11].

For all analyses, reactivation was defined virologically as any positive PCR detection, without a viral-load threshold or additional clinical criteria (HSV-1: any positive PCR in BALF; CMV: any positive PCR in whole blood). Institutional standard operating procedures recommended HSV-1 treatment in patients with viral loads exceeding 10⁵ copies/mL in BALF in combination with respiratory deterioration and clinical or radiological signs of pneumonia, at the discretion of the treating physician. When treatment was initiated, the institutional standard suggested intravenous acyclovir at a dose of 5 mg/kg every 8 hours for 10 days, with dose adjustment to 5 mg/kg twice daily in patients undergoing continuous veno-venous hemodialysis. For CMV reactivation, intravenous ganciclovir was recommended, with dose adjustment according to renal function. In patients with immunosuppression, the institutional standard recommended prompt treatment initiation after HSV-1 or CMV PCR detection, whereas in patients without immunosuppression, treatment initiation was based on the clinical judgement of the consultant in charge. Since the publication of the RECOVERY trial in July 2020, all patients with SARS-CoV-2 pneumonia received dexamethasone at a dose of 6 mg per day, administered orally or intravenously, for a duration of 10 days [12]. Veno-venous extracorporeal membrane oxygenation (vvECMO) (Cardiohelp, Getinge, Rastatt, Germany) was initiated according to the Extracorporeal Life Support Organization guidelines [13].

Data were obtained from the hospital’s digital patient records and associated databases (CGM Medico, CompuGroup Medical SE & Co. KGaA, Koblenz). All available clinical data, including laboratory values, microbiological findings, and virological findings, were included in the analysis. The study was conducted in accordance with the Declaration of Helsinki and was approved by the ethics committee (approval number: 24–12239-BO) of the Medical Faculty, University of Duisburg-Essen, Germany. The manuscript adheres to the *STROBE Statement* – checklist of items that should be included in reports of observational studies (**Supplementary** S1 Table). The study was registered in the Deutsches Register Klinischer Studien (DRKS-ID DRKS00038751).

The primary endpoint was to evaluate independent risk factors influencing ICU mortality. The secondary endpoints were 30-day mortality, the crude incidence of HSV-1, CMV, organ failure, need for invasive mechanical ventilation and need for vvECMO support. Categorical variables were presented as absolute numbers and percentages. Continuous variables were presented as mean ± standard deviation or median (interquartile range) if non-normally distributed following assessment by the Shapiro-Wilk test. Analysis was conducted using the chi-squared test for categorical variables and the two-sided Student’s t-test or Mann–Whitney U test for continuous variables. Statistical significance was defined as p < 0.05. Univariable analysis was conducted using logistic regression for ICU survival. Confounding variables were assessed by multivariable analysis. Survival was analyzed using Kaplan–Meier analysis and the log-rank test. As a sensitivity analysis addressing the time-dependent nature of reactivation, HSV-1 status was additionally modeled as a time-dependent covariate in a Cox proportional-hazards model (counting-process formulation), with patients considered exposed from the day of first HSV-1 detection onwards. Statistical analysis was performed using the software package R, version 4.5.1 (The R Foundation).

## Results

### Patient characteristics and baseline data

Following screening, 455 patients were included in the final analysis. The mean age was 57 years (range: 13–94 years), and 316 (69%) were male. The median follow-up was 14 days (range: 0–143), and the median length of ICU stay was 14 days (range: 0–90). COVID-19 pneumonia was the most common primary reason for ICU treatment, accounting for 423 (93%) patients, followed by postoperative surveillance after major surgery in 33 (7%) patients with concomitant COVID-19 without respiratory symptoms. Of the 455 patients, 260 (57%) were secondary referrals from external hospitals.

### Incidence and characteristics of patients with herpesvirus reactivation

**Fig 1:**
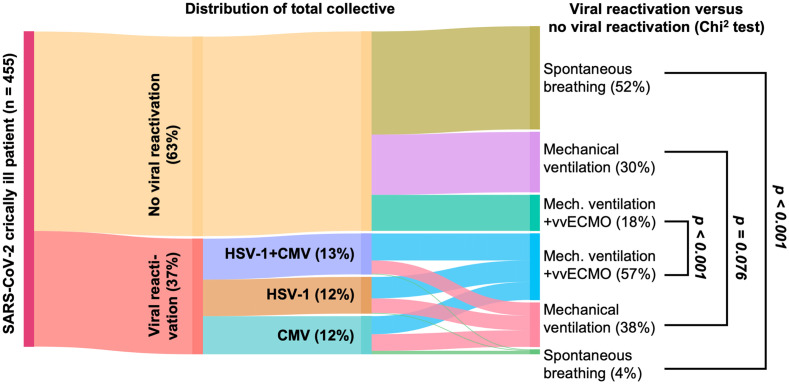
Incidence of viral detection and associated clinical outcomes. Alluvial plot depicting the incidence of viral detection in the study cohort, stratified by virus type and selected clinical endpoints. Flows connect the initial groups to the clinical outcomes shown on the right. The width of each flow is proportional to the number of patients in each subgroup. Colors represent viral categories and illustrate their contribution to the different clinical outcomes. Statistical comparisons were performed using the chi-square test, with p-values indicated on the right. CMV, cytomegalovirus; HSV-1, herpes simplex virus type 1; vvECMO, veno-venous extracorporeal membrane oxygenation.

**Fig 2:**
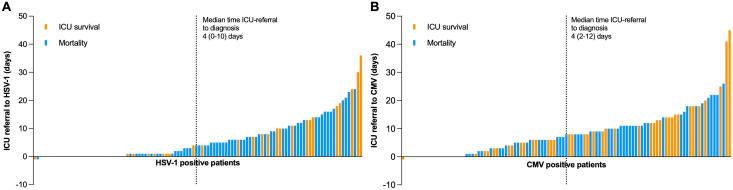
Timing of viral detection. Waterfall plots showing the time from ICU admission to viral detection in individual patients for **A**, herpes simplex virus type 1 (HSV-1) and **B**, cytomegalovirus (CMV). Each bar represents one patient. Blue bars indicate patients with mortality, whereas orange bars indicate ICU survivors.

Of the 455 patients included, 164 (36%) developed viral reactivation with HSV-1, CMV, or both, during their ICU stay (Fig 1). Isolated HSV-1 reactivation was observed in 54 (12%) patients, of whom 53 (98%) received antiviral therapy with acyclovir. Isolated CMV reactivation occurred in 53 (12%) patients, with ganciclovir administered in 9 cases. Among the 57 (13%) patients with combined CMV and HSV-1 reactivation, all received therapy with acyclovir and 13 (23%) additionally received CMV treatment with ganciclovir. The median duration from admission to ICU to viral detection was 4 (0−10) days for HSV-1 and 4 (2−12) for CMV, respectively (Fig 2). The median age was identical in both groups (reactivation of CMV, HSV-1, or both compared with no additional virus besides SARS-CoV-2) at 57 years (p = 0.4). The sex distribution was comparable, with 69% male and 31% female patients in both groups (p > 0.9), as was the mean body mass index of 29 kg/m² (p = 0.8). A difference was observed in the occurrence of pulmonary embolism, which was markedly more frequent in patients with viral reactivation (HSV-1, CMV, or both) than in those without reactivation (24% vs 10%; p < 0.001). Viral reactivation was more common in patients with secondary referral from external hospitals (48% vs 20%; p < 0.001).

Patients with viral reactivation (HSV-1, CMV, or both) were more likely to be invasively mechanically ventilated (96% vs 49%; p < 0.001) and had a substantially longer duration of invasive mechanical ventilation (509 vs 112 h; p < 0.001). The initial Horowitz index (PaO₂/FiO₂ ratio) was lower in the reactivation group than in the non-reactivation group (115 vs 135 mmHg; p = 0.005). In those without invasive mechanical ventilation, non-invasive ventilation (NIV) was the most common respiratory support modality (81%). vvECMO therapy was more frequently required among patients with viral reactivation (57% vs 18%; p < 0.001), and the duration of vvECMO support was longer (362 vs 114 h; p < 0.001). Sepsis occurred more often in patients with viral reactivation (HSV-1, CMV, or both) (78% vs 37%; p < 0.001). Renal replacement therapy was also more frequent (54% vs 25%; p < 0.001), and its duration was significantly longer (184 vs 70 h; p < 0.001). ICU length of stay was significantly prolonged in patients with viral reactivation (20 vs 6 days; p < 0.001), 30- and 90-day mortality were significantly higher (49% vs 33%; p < 0.001). The baseline characteristics of patients with and without viral reactivation are shown in detail in Table 1.

**Table 1:** Baseline characteristics of all included patients.

Characteristic	Patients without viral reactivation (n = 291)	Patients with HSV/CMV reactivation (n = 164)	p-value
Age (years)	57 (54 - 58)	57 (53 - 57)	0.4
Sex (%), male female	69 31	69 31	> 0.9
BMI (kg/m^2^)	29 (30 - 32)	29 (30 - 32)	0.8
SAPS initial	30 (30 - 34)	36 (34 - 37)	< 0.001
CAD (%)	16 (12 - 21)	8.4 (4.8 - 14)	0.021
CRP initial (mg/dL)	13 (14 - 17)	17 (16 - 19)	0.05
HSV copies/ mL		492.000 (4.252 - 2.400.000)	
CMV copies/ mL		299 (116 - 2.187)	
Sepsis (%)	37 (31 - 43)	78 (71 - 84)	< 0.001
Pulmonary embolism (%)	10 (7.3 - 15)	24 (18 - 31)	< 0.001
No oxygen (%)	2.8 (1.4 - 5.3)	0	
Low-flow nasal oxygen (%)	3.1 (1.6 - 5.8)	0	
Oxygen via face mask (%)	2.8 (1.4 - 5.3)	0	
High-flow nasal oxygen (%)	0.7 (0.1 - 2.5)	0	
NIV (%)	42 (36 - 47)	3.7 (1.7 - 7.8)	< 0.001
Invasive mechanical ventilation (%)	49 (43 - 55)	96 (91 - 98)	< 0.001
Horowitz index (mmHg)	135 (158 - 186)	115 (129 - 158)	0.005
vvECMO therapy (%)	18 (14 - 23)	57 (50 - 65)	< 0.001
CVVHDF (%)	25 (20 - 31)	54 (47 - 62)	< 0.001
Solid organ transplantation	2.8 (1.4 - 5.3)	2.4 (1.0 - 6.1)	0.843
Dexamethasone	73 (68 - 78)	80 (74 - 86)	0.081
T-cell immunosuppression (prior 90 days; any)	4.8 (2.9 - 7.9)	4.9 (2.5 - 9.3)	0.975
B-cell immunosuppression (any)	4.1 (2.4 - 7.1)	4.3 (2.1 - 8.5)	0.941
Tocilizumab	6.9 (4.5 - 10.4)	6.1 (3.3 - 10.9)	0.749
Inborn immunodeficiency	0	0.6 (0.0 - 3.4)	

Continuous variables are presented as median (95% confidence interval (CI)). Categorical variables are presented as percentage (95% CI). BMI, body-mass-index; CAD, coronary artery disease; CRP, c-reactive protein; CVVHDF, continuous veno-venous hemodiafiltration; NIV; non-invasive ventilation; SAPS, simplified acute physiology score; vvECMO, veno-venous extracorporeal membrane oxygenation, NLR, neutrophil-lymphocyte ratio.

Peak CRP levels were higher in patients with than without reactivation (30 vs 20 mg/dL; p < 0.001). Similarly, peak leucocyte counts were markedly elevated (24,000 vs 15,000 cells/µL; p < 0.001).

### Patients with isolated HSV-1 reactivation

Of the 164 (36%) patients who developed viral reactivation, 54 (33%) showed isolated HSV-1 reactivation in BALF. The median age in this subgroup was 58 years, and the majority was male (58 years with reactivation vs 57 years without reactivation; p = 0.8, and 70% vs 69%; p > 0.9, respectively). Pulmonary embolism was observed more than twice as often in patients with isolated HSV-1 reactivation compared with those without any herpesvirus reactivation (27% vs 10%; p = 0.003).

Patients with isolated HSV-1 reactivation required invasive mechanical ventilation more than twice as frequently, and the duration of invasive mechanical ventilation was more than three times longer than in patients without viral reactivation (98% vs 48%; p < 0.001, and 463 vs 112 h; p < 0.001, respectively). The initial Horowitz index (PaO₂/FiO₂ ratio) was lower in patients with than without HSV-1 reactivation (107 vs 135 mmHg; p = 0.031). vvECMO support was required more than three times as often and had to be maintained for more than twice as long compared with patients without HSV-1 reactivation (58% vs 18%; p < 0.001, and 311 vs 114 h; p = 0.009, respectively). Secondary referral patients had a higher rate of isolated HSV-1 reactivation (15% vs 7.7%; p = 0.017), with a subsequently higher incidence of vvECMO therapy (72% vs 20%; p < 0.001) compared with patients admitted directly or transferred internally. Renal replacement therapy was administered more than twice as frequently in patients with than without HSV-1 reactivation (57% vs 25%; p < 0.001) and was maintained for a longer duration (193 h vs 70 h; p = 0.003).

Sepsis was described more often in patients with isolated HSV-1 reactivation (79% vs 37%; p < 0.001). ICU length of stay was longer in patients with than without HSV-1 reactivation (19 vs 6 days; p < 0.001). Thirty-day mortality was markedly increased in patients with than without HSV-1 reactivation (60% vs 33%; p < 0.001). **Supplementary** S2 Table shows the characteristics of patients with isolated HSV-1 reactivation. Among the 54 patients with isolated HSV-1 reactivation, 53 (98%) received antiviral therapy, whereas only one patient did not.

### Patients with isolated CMV reactivation

Among the 164 patients with viral reactivation, 53 (32%) were positive exclusively for CMV. The median age was 56 years (compared with 57 years in patients without any reactivation; p = 0.6), and the majority were male (71%; p = 0.9). The median CMV viral load in whole blood were 413 IU/ml.

Secondary referral patients had a higher rate of isolated CMV reactivation (16% vs 6.2%; p = 0.002) compared with patients admitted directly or transferred internally. In cases of isolated CMV reactivation, invasive mechanical ventilation occurred more frequently and lasted longer than in patients without viral reactivation (91% vs 48%; p < 0.001, and 437 vs 112 h; p < 0.001, respectively). In patients with isolated CMV reactivation, vvECMO therapy was required more often and for a longer duration (47% vs 18%; p < 0.001, and 338 vs 114 h; p = 0.003, respectively) compared with patients without viral reactivation. Renal replacement therapy was more frequent in patients with isolated CMV reactivation than in those without viral reactivation (45% vs 25%; p = 0.003). Patients with isolated CMV reactivation also developed sepsis considerably more often (69% vs 37%; p < 0.001). Peak leucocyte, neutrophil, and lymphocyte counts were higher in patients with isolated CMV reactivation than in those without CMV reactivation (22/nL vs 15/nL; p < 0.001; 17/nL vs 11/nL; p < 0.001; and 1.97/nL vs 1.53/nL; p = 0.049, respectively). CMV viral loads in whole blood differed significantly between treated and untreated groups with 25.561 copies IU/ml (median) in the treated and 304 copies IU/ml (median) in the untreated group (p = 0.04). Among treated patients, four of nine (44%) were immunosuppressed. Two of the four immunosuppressed patients with CMV reactivation died, whereas one of five non-immunosuppressed patients died during their ICU stay. Thirty-day mortality did not differ between patients with and without CMV reactivation (33% vs 38%; p = 0.5); however, ICU length of stay was markedly prolonged in those with CMV reactivation (18 vs 6 days; p < 0.001). Supplementary S3 Table shows the characteristics of patients with isolated CMV reactivation.

### ICU survival

The univariable analysis identified age as a strong predictor of ICU survival (Table 2). Each additional year of age was associated with a 3% stepwise reduction in ICU survival (OR 0.97, p < 0.001). Markers of disease severity and organ support were predictive of survival: vvECMO requirement (OR 0.12, p < 0.001), invasive mechanical ventilation (OR 0.10, p < 0.001) and CVVHDF (OR 0.06, p < 0.001) were all independently associated with reduced odds of survival, reflecting the impact of multi-organ dysfunction. Higher inflammatory markers including peak procalcitonin (OR 0.98, p < 0.001) and peak CRP (OR 0.94, p < 0.001) were associated with lower survival. Confirmation of sepsis as per established criteria decreased the odds of survival (OR 0.33, p < 0.001). Dexamethasone therapy was applied in 345 (76%) of patients and was not associated with survival in our collective (OR 0.93, p = 0.737). Further forms of immunosuppression were rare (e.g., solid organ transplantation 2.6%, T-cell-suppressing medication 4.8%, tocilizumab 6.9%) and therefore not included. In the multivariable logistic regression analysis of ICU survival (Table 3), increasing age was associated with a lower probability of survival (odds ratio: 0.95; p < 0.001). vvECMO therapy (OR 0.08, p < 0.001) and renal replacement therapy (dialysis, CVVHDF; OR 0.06, p < 0.001) were independently associated with reduced survival.

**Table 2:** Univariable analysis of ICU survival.

Variable	Odds ratio (95% CI)	p-value
**Age**	0.97 (0.96 - 0.98)	< 0.001
**vvECMO therapy**	0.12 (0.08 - 0.19)	< 0.001
**vvECMO therapy (h)**	0.76 (0.65 - 0.87)	< 0.001
**Invasive mechanical ventilation (any)**	0.10 (0.06 - 0.17)	< 0.001
**Invasive mechanical ventilation (h)**	0.89 (0.85 - 0.94)	< 0.001
**CVVHDF**	0.06 (0.04 - 0.10)	< 0.001
**CRP (mg/dL)**	0.94 (0.93 - 0.96)	< 0.001
**PCT (ng/mL)**	0.98 (0.98 - 0.99)	< 0.001
**Sepsis**	0.33 (0.22 - 0.48)	< 0.001
**HSV-1**	0.27 (0.17 - 0.44)	< 0.001
**CMV**	0.50 (0.32 - 0.77)	0.002
**Baseline neutrophil count (×10⁹/L)**	0.95 (0.93 - 0.97)	< 0.001
**Baseline lymphocyte count (×10⁹/L)**	1.11 (1.07 - 1.15)	< 0.001
**NLR**	0.94 (0.92 - 0.96)	< 0.001

Associations are expressed as odds ratios (OR) with 95% confidence intervals (CI) and corresponding p-values, derived from univariable binary logistic regression with ICU survival as the outcome; an OR below 1 indicates an association with reduced survival (i.e., increased mortality). Continuous variables are modeled per one-unit increase on their natural scale (age per year; NLR, neutrophil and lymphocyte counts per unit). CMV, cytomegalovirus; CRP, c-reactive protein; CVVHDF, continuous veno-venous hemodiafiltration; HSV-1, herpes simplex virus 1; PCT, procalcitonin; NLR, neutrophil-lymphocyte ratio; vvECMO, veno-venous extracorporeal membrane oxygenation.

**Table 3:** Multivariable analysis of ICU survival.

	Odds ratio (95% CI)	p-value
**Age**	0.95 (0.93 - 0.97)	< 0.001
**vvECMO therapy**	0.08 (0.03 - 0.17)	< 0.001
**Invasive mechanical ventilation (h)**	1.25 (1.12 - 1.39)	< 0.001
**CVVHDF**	0.06 (0.03 - 0.12)	< 0.001
**HSV-1**	0.35 (0.17 - 0.74)	0.006
**NLR**	0.97 (0.94 - 0.99)	0.011

Associations are expressed as odds ratios (OR) with 95% confidence intervals (CI) and corresponding p-values, derived from a multivariable binary logistic regression model with ICU survival as the outcome; an OR below 1 indicates an association with reduced survival (i.e., increased mortality). All continuous predictors are expressed per one-unit increase on their natural scale (age per year), consistent with the univariable analysis (Table 2); estimates therefore differ from the standardized values reported in the original submission, while the underlying model and conclusions are unchanged. CVVHDF, continuous veno-venous hemodiafiltration; HSV-1, herpes simplex virus 1; NLR, neutrophil-to-lymphocyte ratio; vvECMO, veno-venous extracorporeal membrane oxygenation.

**Fig 3:**
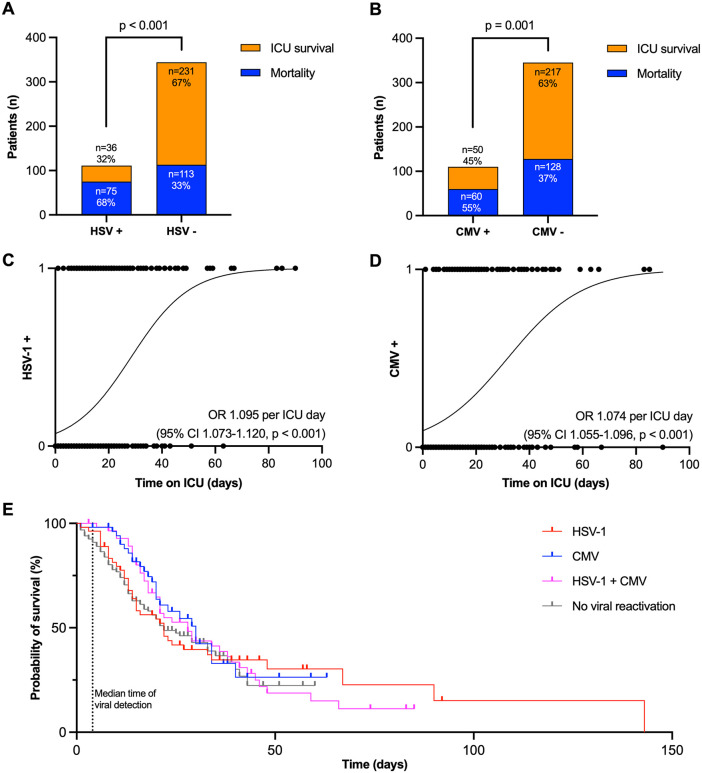
Viral detection and ICU survival. **A-B**, stacked bar plot showing patients with and without viral detection and distribution of ICU survival and mortality. Statistical testing was conducted with Chi^2^ test. **A**, herpes simplex virus type 1 (HSV-1), **B**, cytomegalovirus (CMV; each combine isolated reactivation and co-reactivation). **C-D**, logistic regression analysis of viral detection correlated to the time-point of viral detection relative to ICU admission for HSV-1 and CMV, respectively. **E**, Kaplan Meier analysis of mortality, stratified by group of viral detection. The median time from ICU admission to first viral detection is indicated. Logrank test was nonsignificant.

HSV-1-positive patients exhibited higher mortality (rate) compared to HSV-1-negative patients (68% vs 33%; χ² = 41.72, p < 0.001), corresponding to an unadjusted odds ratio of 4.26 (95% CI 2.67–6.74) (Fig 3). CMV-positive patients also showed increased mortality (rate) compared to CMV-negative patients (55% vs 37%; χ² = 10.47, p = 0.002), although the effect size was less pronounced (OR 2.03, 95% CI 1.31–3.11). Longer ICU stay was strongly associated with an increased likelihood of HSV-1 detection (OR 1.10 per additional ICU day, 95% CI 1.07–1.12, p < 0.001), corresponding to a 57% increase in odds per 5-day prolongation of ICU stay. A similar but less pronounced association was observed for CMV detection (OR 1.07 per ICU day, 95% CI 1.06–1.10, p < 0.001). Both models demonstrated good discrimination (area under the curve (AUC) 0.82 for HSV-1 and 0.82 for CMV). In multivariable analysis adjusting for confounders, detection of HSV-1 remained associated with reduced ICU survival (OR 0.35; p = 0.006). CMV was not associated with ICU survival in multivariable analysis. Log-rank analysis of survival from ICU admission did not reveal significant differences, likely reflecting immortal time bias due to the time-dependent nature of viral detection. To address this, HSV-1 was modeled as a time-dependent covariate in a Cox proportional-hazards model, in which HSV-1 reactivation remained associated with increased ICU mortality (hazard ratio (HR) 1.56, 95% CI 1.14–2.14, p = 0.006), whereas a time-fixed model did not (HR 1.08, p = 0.623), consistent with an immortal-time-bias effect in the naïve analysis (Supplementary S4 Table).

**Fig 4:**
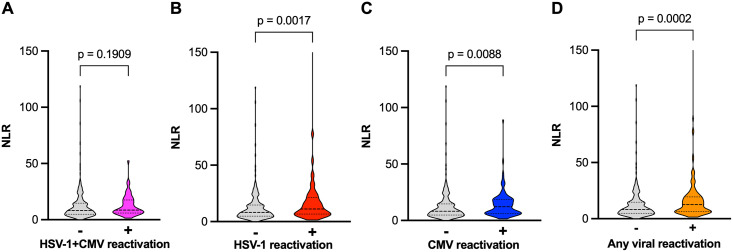
Neutrophil-lymphocyte ratio (NLR). Violin plots depicting NLR with **A**, concomitant detection of herpes simplex virus type 1 (HSV-1) and cytomegalovirus (CMV), **B**, HSV-1-only, **C**, CMV-only and **D**, any viral detection, respectively. NLR was derived from first laboratory analysis following admission to ICU. The long-dashed line indicates the median, and the short-dashed lines indicate the first and third quartile.

The initially measured lymphocyte proportion was significantly lower in patients with than without viral reactivation (7% vs 10%; p < 0.001). The neutrophil-lymphocyte ratio (NLR) was increased in patients with additional viral reactivation, including HSV-1 (11.2 versus (vs) 8.2), CMV (12.2 vs 8.2) and any viral detection (12.5 vs 8.2; Fig 4). NLR correlated with reduced survival in univariable analysis (odds ratio 0.94 (0.92–0.96); p < 0.001) with a small absolute effect size. Receiver operating characteristics determined an AUC of 0.609 and 0.589 for HSV-1 and CMV, respectively (Supplementary S1 Fig). The detection of HSV-1 and an elevated NLR remained associated with a lower probability of survival after multivariable adjustment (OR 0.35; p = 0.006 and OR 0.97; p = 0.011 respectively), albeit with a small effect size per unit increase in NLR. The HSV-1 viral load (copies/ mL BALF) was not associated with ICU survival, neither in univariable logistic regression (OR 1.00, 95% CI 1.00–1.00, p = 0.825) nor when comparing ICU survivors and non-survivors (median 774,710 vs 492,000 copies/mL; p = 0.654) or in a Cox proportional-hazards model using the log₁₀-transformed load (HR 0.91 per log₁₀ increase, 95% CI 0.77–1.07, p = 0.230). This absence of a dose-response relationship is likely accentuated by the distribution of measured loads, a substantial proportion of which reached the upper limit of assay quantification.

## Discussion

The key finding of this study is that herpesvirus reactivation, particularly HSV-1, is common in critically ill adult patients with COVID-19 in the ICU and is associated with an increased need for organ support, higher rates of sepsis, prolonged ICU stay, and reduced survival.

### Herpes simplex virus type 1 (HSV-1)

Overall, 36% of patients included in our study developed viral reactivation with HSV-1, CMV, or both, during their ICU stay. Other studies of critically ill patients with COVID-19 have reported a wide variation in the prevalence of HSV-1 reactivation, ranging from 25% to 80% [14,15], however with substantially smaller collectives. The marked heterogeneity between studies is likely explained by differences in the definitions of viral reactivation and patient populations of the participating centers. Some studies define any positive HSV-1 PCR as reactivation, while others use distinct thresholds or clinical signs of manifest infection [6,16,17]. Disease severity is another important determinant. As a tertiary referral center, our institution treated a high proportion of secondary referrals, among whom vvECMO use was markedly higher than in primary admissions (47% vs 13%; p < 0.001). Differences in disease severity may have contributed to variations across comparable studies, impacting the incidence of viral reactivation and its association with adverse outcomes. High heterogeneity in HSV-1 prevalence data in European countries must be considered further, ranging from 67.2% in northern Europe to 81.4% in southern Europe [18].

In our study, HSV-1 was associated with organ dysfunction (renal replacement therapy, invasive mechanical ventilation, sepsis, and vvECMO), longer ICU stay, and reduced survival compared to patients without viral reactivation. A multivariable analysis determined HSV-1 to be associated with reduced survival. Correspondingly, most previous studies have also reported a higher burden of disease in COVID-19 patients with herpesvirus reactivation, such as prolonged invasive mechanical ventilation [7] or extended ICU stay [6,7,19]. Meyer et al. reported higher ICU mortality in patients with HSV-1 reactivation (52.5%) than in those without reactivation (31.9%), however in a substantially smaller collective [6]. They also observed an increased risk of mortality and pneumonia in patients with HSV-1 reactivation detected in blood, but not in BALF, suggesting that viraemia may play a more decisive role.

While 53 of 54 (98%) of patients with isolated HSV-1 reactivation received antiviral therapy in our collective, only 70% of patients in Meyer et al. [6] received acyclovir (collective: n = 153 patients). In other studies, between 33% and 80% received antiviral therapy [6,7,19,20], without robust evidence on a beneficial effect on outcome. Due to the lack of randomized prospective data, it remains unknown whether antiviral therapy confers a survival benefit, considering that HSV-1 reactivation may represent a marker of impaired immune status rather than a direct pathogenic driver in some cases. Critical factors include dose adjustment and toxicity, both insufficiently characterized by the available evidence to date [21–23]. This is particularly relevant in critically ill patients requiring renal replacement therapy, which affected more than half of our cohort (e.g., 30 (56%) of patients with isolated HSV-1 reactivation) and substantially influences acyclovir dosing and pharmacokinetics. Most studies of critically ill patients with COVID-19 and HSV-1 reactivation do not provide detailed information on acyclovir dosing or dose adjustment according to renal function or renal replacement modality, highlighting an important gap in the available evidence [6–8,19,20].

### Cytomegalovirus (CMV)

In our cohort, 53 of the 164 patients (32%) with viral reactivation showed exclusive CMV reactivation in BALF or blood, of whom only 9 (17%) received antiviral therapy. In prior studies, reported rates range from 10.0% to 25.4% [15,24]. A major driver includes the use of plasma versus whole blood, since viral counts are higher in whole blood, as applied in our study. In a study by Reizine et al. [24] including 122 patients with COVID-19-related ARDS, 10% showed CMV reactivation, defined as any detection of CMV in blood by PCR. Yamamoto et al. [15] not only considered CMV DNA detection in blood but also proposed a clinical definition of CMV disease (proven, possible, and probable). In that cohort, 25.4% of patients with severe COVID-19 had CMV infection, whereas only 10.2% received treatment. In our study, most CMV-positive patients were IgG-positive and IgM-negative, indicating latent virus reactivation rather than primary infection. This finding is consistent with other reports and may explain the relatively milder clinical phenotype [16,25]. López-Olivencia et al. [26] reported higher ICU mortality among COVID-19 patients with CMV reactivation (43.8% vs 13.6%; p < 0.001). Niitsu et al. [27] also observed a marked difference in mortality between patients with and without CMV reactivation (33% vs 0%; p < 0.046). Similarly, Luyt et al. [17] showed that CMV reactivation in BALF, defined by PCR positivity, was not associated with increased mortality in patients with COVID-19.

In the cohort assessed here, CMV detection was largely limited to low-level viremia, with a median viral load of 413 IU/mL, affecting only a small proportion of patients. CMV viral loads differed between treated and untreated groups (ganciclovir-treated patients: 25.561 IU/ml median vs 305 IU/ml in untreated patients (p = 0.04)). Importantly, there was no evidence of end-organ disease (e.g., colitis, pneumonitis), indicating that these cases did not meet criteria for clinically significant CMV disease. Low rates of antiviral treatment may have further been driven by the fact that CMV reactivation in our study was defined as any detection of CMV DNA in whole blood, whereas treatment decisions were based on clinical judgement rather than solely on viral copy numbers. In this context, CMV reactivation in this cohort may represent a bystander phenomenon rather than a driver of disease severity, which could explain why CMV co-detection did not worsen outcomes compared with HSV reactivation alone. CMV may function more as a surrogate marker of immune suppression or critical illness rather than as a direct pathogenic driver [28,29].

### Neutrophil-lymphocyte ratio (NLR)

Inflammatory markers, including peak CRP and leucocyte counts, were substantially higher in patients with viral reactivation. This has also been reported by other study groups [8,30], including a recently published meta-analysis [31]. We therefore conclude that the elevated inflammatory markers reflect a greater burden of disease in these patients [31]. Patients from our collective with HSV-1 and CMV reactivation showed higher neutrophil counts and lower lymphocyte proportions. Lymphopenia may promote adverse outcomes in critically ill patients with COVID-19, reflecting pronounced immune dysregulation, as was already observed early during the pandemic [32,33]. Other groups, however, interpret lymphopenia in COVID-19 primarily as a marker of disease severity and more advanced organ dysfunction [34]. Sepsis-associated endothelial injury, barrier disruption, and enhanced leucocyte adhesion and extravasation may further contribute to lymphocyte depletion in the most severely ill patients [35].

In the present dataset, patients with HSV-1, CMV or any viral detection (combined group) showed significantly increased NLRs, suggesting a systemic inflammatory response following a state of immunosuppression with absolute lymphopenia in patients with COVID-19 [20,36–38]. Exogenous corticosteroids may be considered major modifiers of systemic immune cell ratios because they exert pleiotropic effects that vary depending on the target and immune cell type [12]. In lymphocytes, they induce apoptosis and alter cellular function [39]. They also suppress inflammatory cytokine production. In patients with severe COVID-19, dexamethasone therapy has been shown to affect circulating neutrophils, including downregulation of interferon-stimulated genes [40]. Franceschini et al. [41] reported higher rates of HSV-1 reactivation in patients with COVID-19 receiving concomitant dexamethasone therapy. Because almost all patients in our cohort received dexamethasone following RECOVERY study results [12], we were unable to assess its specific impact on herpesvirus reactivation.

### Limitations

The study’s retrospective, single-center design limits causal interpretation and the generalizability of the results. Viral reactivation was defined using virological detection without direct clinical correlation, limiting discrimination between colonization, reactivation, and invasive infection. Treatment with corticosteroids represented a potential confounding factor. The high proportion of secondary referrals for vvECMO therapy introduced a risk of referral bias through the preferential inclusion of patients with the most severe disease. The dependence of outcome measures on ICU length of stay may have introduced immortal time bias: patients with more severe disease died early, thereby having less opportunity to reach certain endpoints, whereas patients with less severe disease survived longer and accumulated these outcomes, potentially leading to a spurious association suggesting a more favorable effect, an association that persisted in a time-dependent Cox sensitivity analysis but requires prospective validation.

## Conclusion

Herpesvirus reactivation was common in critically ill patients with COVID-19 and was associated with greater disease severity, increased need for organ support, and reduced ICU survival. HSV-1 detection was associated with ICU mortality, supporting systematic viral monitoring and prospective studies to define clinically meaningful thresholds for antiviral intervention.

## Supporting information

S1 Fig Neutrophil-lymphocyte ratio (NLR). **A**, receiver operating characteristics of HSV-1 + CMV, HSV-1-only, CMV-only and any viral reactivation. AUC. Area under the curve. **B**, linear regression analysis of HSV-1 copies and CMV-copies (peak values) with NLR.
(PDF)

S2 Fig Summarizing Figure. This figure summarizes the study population and main results. A total of 455 ICU patients with COVID-19 were included; all patients were routinely tested for herpes simplex virus type 1 (HSV-1) and cytomegalovirus (CMV) reactivation during their ICU stay. Any viral reactivation was linked to higher mortality. HSV-1 reactivation was also associated with increased use of invasive mechanical ventilation, veno-venous extracorporeal membrane oxygenation (ECMO) and renal replacement therapy. Created in BioRender. https://BioRender.com/why5vf5
(JPEG)

S1 Table STROBE Statement – checklist of items that should be included in reports of observational studies. (PDF)

S2 Table Characteristics of patients with isolated HSV-1 reactivation. (PDF)

S3 Table Characteristics of patients with isolated CMV reactivation. (PDF)

S4 Table Sensitivity analysis. (PDF)

## Abbreviations

ARDS: Acute respiratory distress syndrome
BALF: Bronchoalveolar lavage fluid
CMV: Cytomegalovirus
CRP: C-reactive protein
COVID-19: Coronavirus disease 2019
CVVHDF: Continuous veno-venous haemodiafiltration
DRKS: Deutsches Register Klinischer Studien
ECMO: Extracorporeal membrane oxygenation
HSV-1: Herpes simplex virus type 1
ICU: Intensive care unit
IgG: Immunoglobulin G
IgM: Immunoglobulin M
IU: International units
NLR: Neutrophil-lymphocyte ratio
OR: Odds ratio
PCR: Polymerase chain reaction
SOFA: Sequential Organ Failure Assessment
SARS-CoV-2: Severe acute respiratory syndrome coronavirus 2
vvECMO: Veno-venous extracorporeal membrane oxygenation
