# Supplementary figures and images for: Herpesvirus reactivation is associated with mortality in critically ill ICU patients with COVID-19: Insights from a retrospective single-center analysis of 455 cases
Source: PLoS One. 2026 Jul 17;21(7):e0354153. doi: 10.1371/journal.pone.0354153 (PMC13379001; doi:10.1371/journal.pone.0354153)

**A**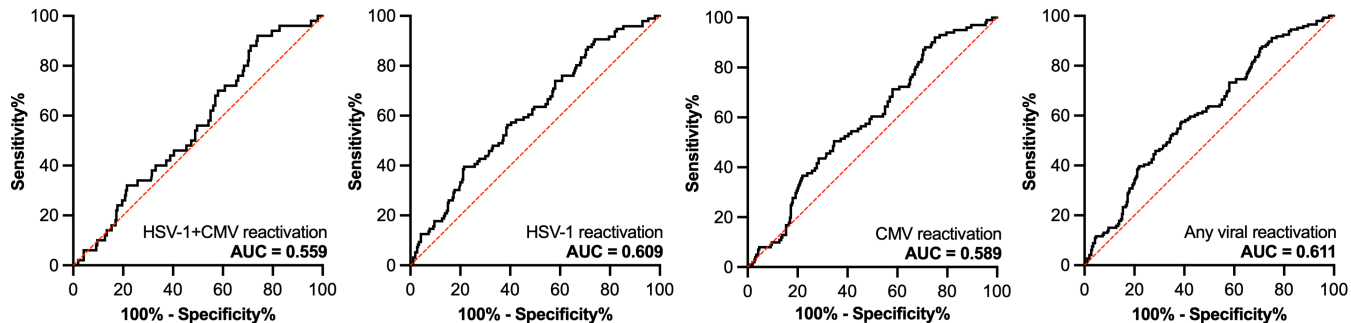**B**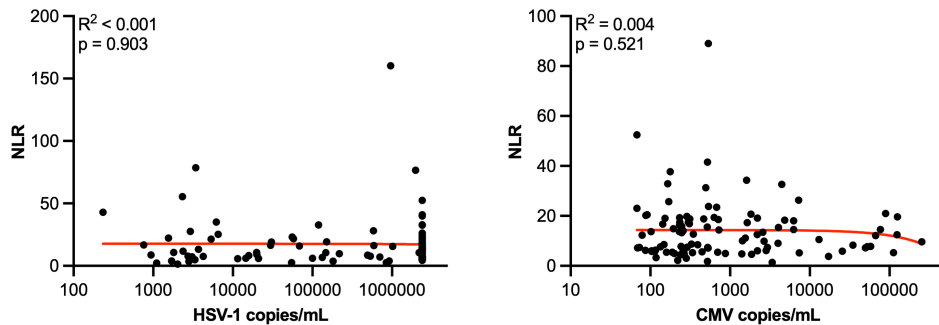

Supplement: S1 Fig — A, receiver operating characteristics of HSV-1 + CMV, HSV-1-only, CMV-only and any viral reactivation. AUC. Area under the curve. B, linear regression analysis of HSV-1 copies and CMV-copies (peak values) with NLR. (PDF) [file pone.0354153.s001.pdf]

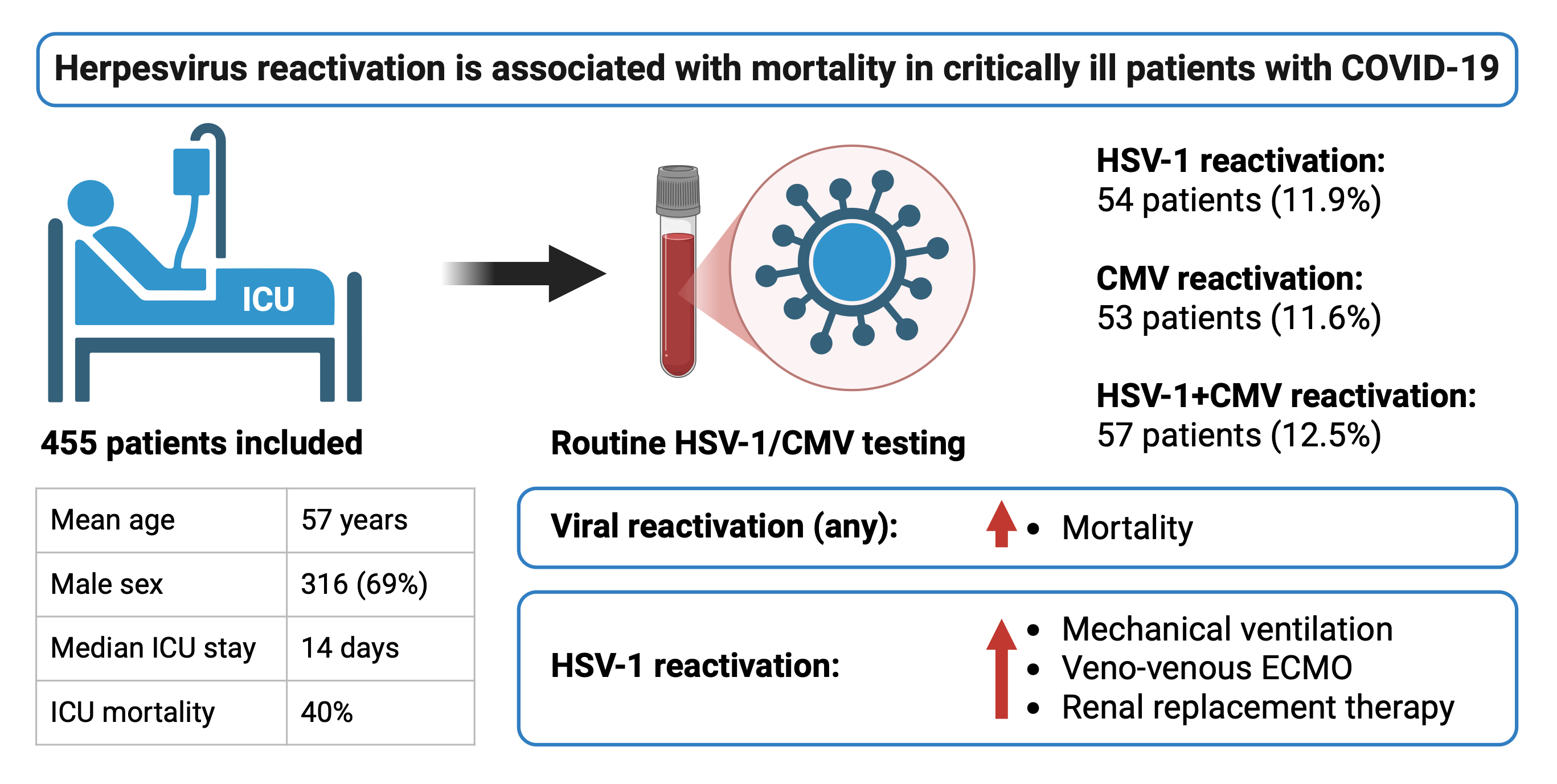

Supplement: S2 Fig — This figure summarizes the study population and main results. A total of 455 ICU patients with COVID-19 were included; all patients were routinely tested for herpes simplex virus type 1 (HSV-1) and cytomegalovirus (CMV) reactivation during their ICU stay. Any viral reactivation was linked to higher mortality. HSV-1 reactivation was also associated with increased use of invasive mechanical ventilation, veno-venous extracorporeal membrane oxygenation (ECMO) and renal replacement therapy. Created in BioRender. https://BioRender.com/why5vf5 (JPEG) [file pone.0354153.s002.jpeg]
