# Supplementary material for: Herpesvirus reactivation is associated with mortality in critically ill ICU patients with COVID-19: Insights from a retrospective single-center analysis of 455 cases
Source: PLoS One. 2026 Jul 17;21(7):e0354153. doi: 10.1371/journal.pone.0354153 (PMC13379001; doi:10.1371/journal.pone.0354153)
Supplement: S2 Table — (PDF) [file pone.0354153.s004.pdf]

Supplementary Table S2. Characteristics of patients with isolated HSV-1 reactivation

Characteristic	Patients without viral reactivation (n = 291)	Patients with isolated HSV-1 reactivation (n = 54)	p-value
**Age (years)**	57 (54 -58)	58 (52 - 65)	0.8
**Gender (%), male**	69	70	> 0.9
**female**	31	30	
**BMI (kg/m^2)**	29 (30 - 32)	31 (29 - 35)	0.6
**SAPS initial**	30 (30 - 34)	38 (34 - 40)	< 0.002
**CAD (%)**	16 (12 - 21)	11 (5 - 24)	0.5
**CRP initial (mg/dL)**	13 (7 - 23)	20 (12 - 26)	0.035
**CRP peak (mg/dL)**	20 (12 - 30)	29 (25 - 35)	0.018
**NLR initial**	8 (5 - 15)	14 (8 - 25)	0.016
**HSV copies/mL**		88.294 (4.371 - 2.400.000)	
**Sepsis (%)**	37 (31 - 43)	79 (66 - 89)	< 0.001
**Pulmonary embolism (%)**	10 (7 - 15)	27 (16 - 41)	0.003
**Intubation (%)**	48 (43 - 54)	98 (89 - 100)	< 0.001
**Invasive ventilation (h)**	112 (168 - 227)	463 (448 - 700)	< 0.001
**Horowitz index (mmHg) initial**	135 (158 -186)	107(110 -166)	0.031
**vvECMO therapy (%)**	18 (14 - 23)	58 (44 - 72)	< 0.001
**vvECMO therapy duration (h)**	114 (137 -239)	311 (246 - 507)	0.009
**CVVHDF (%)**	25 (20 - 31)	57 (42 - 70)	< 0.001

**CVVHDF (h)**	70 (99 - 164)	193 (212 -468)	0.003
**Length of ICU stay (d)**	6 (7 - 9)	19 (18 - 28)	< 0.001
**30-day mortality (%)**	33 (28 - 39)	60 (46 - 73)	< 0.001
**90-day mortality (%)**	36 (31 - 42)	68 (54 - 80)	< 0.001

Continuous variables are presented as median (95% confidence interval). Categorical variables are presented as absolute numbers (n) and percentages (%); BMI, body-mass-index; CAD, coronary artery disease; CVVHDF, continuous veno-venous hemodiafiltration; ICU, intensive care unit, vvECMO, veno-venous extracorporeal membrane oxygenation; SAPS, simplified acute physiology score.
