# Supplementary material for: Herpesvirus reactivation is associated with mortality in critically ill ICU patients with COVID-19: Insights from a retrospective single-center analysis of 455 cases
Source: PLoS One. 2026 Jul 17;21(7):e0354153. doi: 10.1371/journal.pone.0354153 (PMC13379001; doi:10.1371/journal.pone.0354153)
Supplement: S3 Table — (PDF) [file pone.0354153.s005.pdf]

Supplementary Table S3: Characteristics of patients with isolated CMV reactivation

Characteristic	Patients without viral reactivation (n = 291)	Patients with isolated CMV reactivation (n = 53)	p-value
**Age (years)**	57 (54 - 58)	56 (51 - 58)	0.6
**Gender (%), male**	69	71	0.9
**female**	31	29	
**BMI (kg/m^2)**	29 (30 - 32)	29 (28 - 31)	0.2
**SAPS initial**	30 (30 - 34)	35 (32 - 37)	0.069
**CAD (%)**	16 (12 - 21)	4 (1 - 14)	0.011
**CRP initial (mg/dl)**	13 (7 - 23)	18 (13 - 24)	0.029
**CRP peak (mg/dl)**	20 (12 - 30)	30 (25 - 33)	<0.001
**NLR initial**	8 (5 - 15)	14 (6 - 19)	0.13
**CMV copies/mL**		413 (178 - 1.860)	
**Sepsis (%)**	37 (31 - 43)	69 (55 - 80)	<0.001
**Pulmonary embolism (%)**	10 (7 -15)	15 (7 -27)	0.4
**Intubation (%)**	48 (43 - 54)	91 (79 - 97)	< 0.001
**Invasive ventilation (h)**	112 (168 - 227)	437 (406 - 570)	< 0.001
**Horowitz index (mmHg) initial**	135 (158 - 186)	120 (126 -180)	0.2
**vvECMO therapy (%)**	18 (14 - 23)	47 (34 - 61)	< 0.001
**vvECMO therapy duration (h)**	114 (137 - 239)	338 (261 - 447)	0.003
**CVVHDF (%)**	25 (20 - 31)	45 (32 - 59)	0.003

**CVVHDF (h)**	70 (99 - 164)	177 (125 - 325)	0.080
**Length of ICU stay (d)**	6 (7 - 9)	18 (17 - 23)	<0.001
**30-day mortality (%)**	33 (28 - 39)	38 (26 - 52)	0.5
**90-day mortality (%)**	36 (31 - 42)	49 (36 - 63)	0.095

Continuous variables are presented as median (95% confidence interval). Categorical variables are presented as absolute numbers (n) and percentages (%); BMI, body-mass-index; CAD, coronary artery disease; CVVHDF, continuous veno-venous hemodiafiltration; ICU, intensive care unit, vvECMO, veno-venous extracorporal membrane oxygenation; SAPS, simplified acute physiology score.
