# Supplementary material for: Herpesvirus reactivation is associated with mortality in critically ill ICU patients with COVID-19: Insights from a retrospective single-center analysis of 455 cases
Source: PLoS One. 2026 Jul 17;21(7):e0354153. doi: 10.1371/journal.pone.0354153 (PMC13379001; doi:10.1371/journal.pone.0354153)
Supplement: S4 Table — (PDF) [file pone.0354153.s006.pdf]

Supplementary Table S4: Sensitivity analysis

Model	HSV-1 hazard ratio (95% CI)	p-value	Events / n
**Time-dependent covariate (HSV-1 exposure from day of first detection)**	1.56 (1.14–2.14)	0.006	182 / 452
**Time-fixed baseline covariate (for comparison)**	1.08 (0.80–1.47)	0.623	182 / 452

Hazard ratios with 95% confidence intervals (CI) and p-values were derived from Cox proportional-hazards regression with ICU survival time as the outcome (event: ICU death). In the time-dependent model, patients were considered exposed to HSV-1 from the day of first HSV-1 detection onwards (counting-process formulation); the time-fixed model treated HSV-1 status as a baseline covariate and is shown for comparison. Of the 455 patients in the cohort, 3 were excluded from this time-to-event analysis owing to incomplete or inconsistent timing data, leaving 452 patients. CI, confidence interval; HSV-1, herpes simplex virus 1; ICU, intensive care unit.
